# LncRNA SNHG20 promotes migration and invasion of ovarian cancer via modulating the microRNA-148a/ROCK1 axis

**DOI:** 10.1186/s13048-021-00889-8

**Published:** 2021-11-26

**Authors:** Qi Yang, Yu-Jie Dong

**Affiliations:** 1 Department of Obstetrics and Gynecology, China-Japan Union Hospital of Jilin University, No. 126 Xiantai Street, Changchun, 130000 Jilin Province P. R. China; 2 Department of Emergency, China-Japan Union Hospital of Jilin University, Changchun, 130000 P. R. China

**Keywords:** SNHG20, Ovarian cancer, microRNA-148a, ROCK1

## Abstract

**Background:**

Ovarian cancer (OC) is characterized by early metastasis and poor prognosis, which threatens the health of women worldwide. Small nucleolar RNA host gene 20 (SNHG20), a long noncoding RNA (lncRNA), has been verified to be significantly up-regulated in several tumors, including OC. MicroRNA-148a (miR-148a)/rho-kinase1 (ROCK1) axis plays an important role in the modulation of tumor development. However, whether SNHG20 can regulate OC progression through miR-148a/ROCK1 axis remains unclear. Normal human ovarian epithelial cell line and four OC cell lines were adopted for in vitro experiments. Real-time PCR was performed to assess the levels of SNHG20 and miR-148a. OC cell proliferation, apoptosis, invasion and migration were detected using clone formation, flow cytometry, transwell, and wound healing assays, respectively. Tumor xenograft assay was applied to evaluate the effect of SNHG20 on tumor growth in vivo.

**Results:**

Significant higher expression of SNHG20 was observed in OC cell lines. SNHG20 markedly promoted the invasion, migration, proliferation and inhibited the apoptosis of OC cells. SNHG20 enhanced ROCK1 expression by sponging miR-148a, and the direct binding between SNHG20/ROCK1 and miR-148a was identified.

**Conclusion:**

SNHG20 promoted invasion and migration of OC via targeting miR-148a/ROCK1 axis. The present research may provide a novel insight for the therapeutic strategies of OC.

## Background

Ovarian cancer (OC) is the fifth leading death cause for women in the world with the highest mortality rate among different kinds of gynecologic malignancies [1–3]. Because of immune escape, chemoresistance, metastasis, and tumor recurrence, OC patients are still suffering from a low five-year survival rate ranging from 20 to 40%, although many treatments, including radiotherapy, chemotherapy, surgical operation have been greatly improved during the past decades [4–6]. Difficulties in early detection, postoperative recurrence, and remote metastasis may result in the poor prognosis of OC patients [7]. Epigenetic and genetic alterations are considered as contributors to the etiology of OC [8], but their specific pathogenic mechanisms remain unclear. Therefore, identification of the functional molecules involved in the progression and development of OC may provide novel insight into the early diagnosis and treatment for OC.

Long non-coding RNAs (lncRNAs) without protein-coding capacity play a key role in several biological processes through sponging microRNAs (miRNAs) [9–11]. It was reported that some lncRNAs could regulate ovarian cancer progression by regulating miRNAs [12, 13]. A previous report indicated that small nucleolar RNA host gene 20 (SNHG20) was closely correlated with the development of ovarian cancer [6]. As previously reported, SNHG20 promoted OC development via targeting miR-338-3p to increase MCL1 expression [14]. Meanwhile, SNHG20 could promote colorectal cancer cell proliferation, migration and invasion via miR-495/STAT3 axis [15]. In addition, SNHG20 facilitated proliferation, invasion and inhibited apoptosis of lung adenocarcinoma cells via sponging miR-342 to upregulate DDX49 [16]. However, the regulatory role and its related mechanism of SNHG20 in OC need to be further explored.

A previous study suggested that the expression of microRNA-148a (miR-148a) was reduced in OC patients [17]. The significant decreased miR-148a expression was demonstrated to be a predictor for the poor prognosis of OC patients, which was also involved in the regulation of growth and metastasis of OC [18, 19]. In addition, we found the target binding sites between SNHG20 and miR-148a using online Starbase database (http://starbase.sysu.edu.cn/). However, the potential regulatory role between SNHG20 and miR-148a remains unclear. Rho-kinase1 (ROCK1) has been confirmed to be involved in many pathophysiological activities, such as pathogenesis of metabolic-related diseases, cell motility and adhesion, and actin cytoskeleton organization [20]. Besides, the increased expression of ROCK1 has been verified in many types of tumors, which was negatively correlated with the poor prognosis [21]. The higher expression of ROCK1 and its promotion to OC cell metastasis have been identified [2]. Notably, we also found the binding sites between miR-148a and ROCK1 through Starbase database. Meanwhile, miR-148a could suppress the migration and invasion of osteosarcoma cells by inhibiting ROCK1 expression [18]. Therefore, we speculate that SNHG20 might regulate the invasion and migration of OC cells through targeting miR-148a/ROCK1 axis.

In this study, we investigated the aberrant expression of SNHG20 in OC cell lines, and the regulation of SNHG20 in OC cell proliferation and migration. The direct binding between SNHG20/ROCK1 and miR-148a was validated. Finally, whether SNHG20 could regulate the migration and invasion of OC cells through modulating miR-148a/ROCK1 axis was also evaluated. This study may provide a new therapeutic strategy for the prevention and treatment of OC.

## Result

### SNHG20 promoted the proliferation and inhibited apoptosis of OC cells

**Fig. 1:**
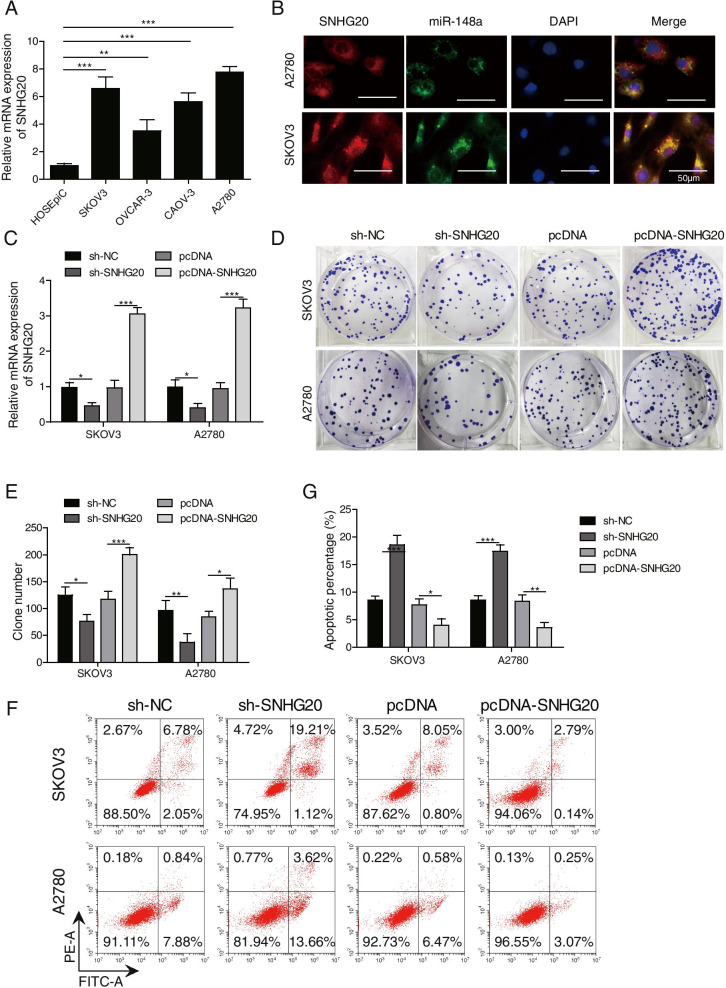
SNHG20 promoted the proliferation and inhibited the apoptosis of OC cells. (**A**) Significant higher mRNA level of SNHG20 in the OC cell lines; (**B**) Measurement of cellular localization of SNHG20 and miR-148a using FISH method; (**C**) SNHG20 level was significantly increased by transfection with pcDNA-SNHG20 and was greatly reduced by transfection with sh-SNHG20; (**D**) The proliferation of OC cells was measured by colony formation assay; (**E**) Quantification analysis of the influence of SNHG20 on cell proliferation; (**F**) The apoptosis of cells was assessed by flow cytometry; (**G**) Quantification analysis of the influence of SNHG20 on cell apoptosis. *P < 0.05, **P < 0.01, ***P < 0.001

To explore the role of SNHG20 in OC development, we firstly examined the differential expression of SNHG20 in OC cells and normal human ovarian epithelial cells. A significant higher expression of SNHG20 was observed in four OC cell lines (SKOV3, OVCAR-3, CAOV-3, and A2780) as compared to normal human ovarian epithelial cell line (Fig. 1A). Furthermore, we measured the cellular localization of SNHG20 and miR-148a using FISH method and found that SNHG20 and miR-148a were mostly located in the cytoplasm of A2780 and SKOV3 cells (Fig. 1B). The SKOV3 and A2780 cell lines with the most significant differential expression of SNHG20 were selected for the transfection with pcDNA-SNHG20 and sh-SNHG20, respectively. The expression of SNHG20 was significantly increased after transfection with pcDNA-SNHG20 (Fig. 1C), while SNHG20 level was greatly declined by sh-SNHG20 (Fig. 1C). We found that overexpression of SNHG20 remarkably promoted proliferation (Fig. 1D-E) and suppressed apoptosis of OC cells (Fig. 1F-G). On the contrary, knockdown of SNHG20 inhibited proliferation (Fig. 1D-E) and induced apoptosis of OC cells (Fig. 1F-G). Therefore, SHNG20 played a key role in regulating the progression of OC.

### SNHG20 promoted the invasion and migration of OC cells

**Fig. 2:**
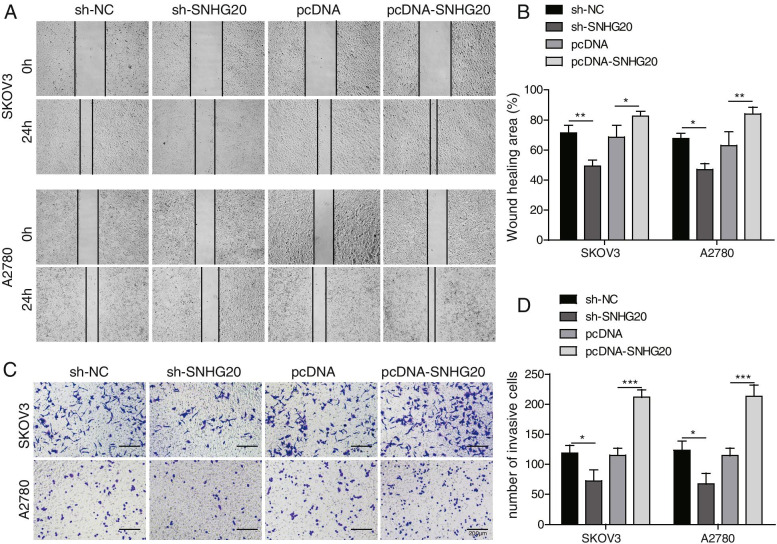
SNHG20 promoted the invasion and migration of OC cells. (**A**) The migration of OC cells was determined by wound healing assay; (**B**) Quantification analysis of the influence of SNHG20 on cell migration; (**C**) The invasion of OC cells was detected by transwell assay; (**D**) Quantification analysis of the influence of SNHG20 on cell invasion. *P < 0.05, **P < 0.01, ***P < 0.001

To uncover the influence of SNHG20 on invasion and migration of OC cells, transwell and wound healing assays were applied. After incubation for 24 h, overexpression of SNHG20 markedly facilitated the migration of OC cells, however sh-SNHG20 exerted an inhibitory effect on migration (Fig. 2A-B). Similar findings were observed in the transwell assay for evaluating invasion. Overexpression of SNHG20 promoted OC cell invasion, whereas knockdown of SNHG20 inhibited invasion (Fig. 2C-D). Therefore, overexpression of SNHG20 could promote OC cell migration and invasion.

### SNHG20 up-regulated ROCK1 level by sponging miR-148a

**Fig. 3:**
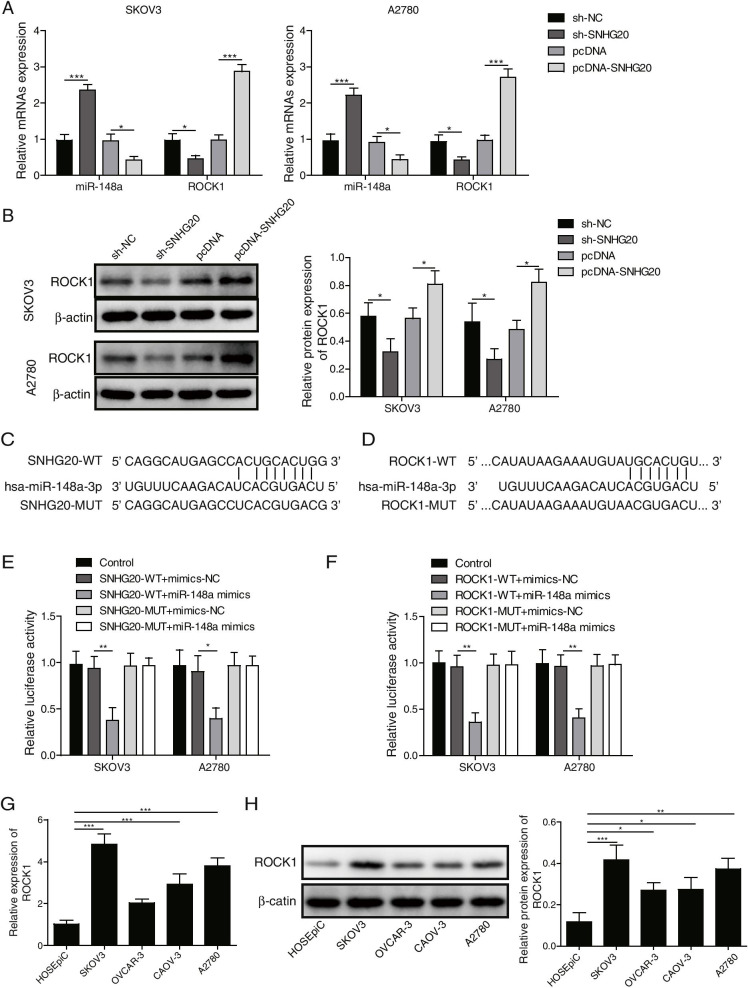
SNHG20 up-regulated ROCK1 level by sponging miR-148a. (**A**) The expression of ROCK1 and miR-148a was measured by qPCR; (**B**) The protein level of ROCK1 was detected by Western blotting; (**C**) Predicted binding site between miR-148a and SNHG20; (**D**) Predicted binding site between miR-148a and ROCK1; (**E**) The direct binding between miR-148a and SNHG20 was validated by dual luciferase assay; (**F**) The direct binding between miR-148a and ROCK1 was validated by dual luciferase assay. (**G**) The protein expression of ROCK1 in the normal ovarian epithelial cells and OC cells was measured by Western blotting. (**H**) Significant higher level of ROCK1 was found in OC cells. *P < 0.05, **P < 0.01, ***P < 0.001

To assess the involvement of miR-148a and ROCK1 in SNHG20-mediated regulation in OC development, we detected the influence of SNHG20 on the expression levels of miR-148a and ROCK1. Overexpression of SNHG20 significantly increased the expression of ROCK1, while decreased the level of miR-148a (Fig. 3A-B). However, we obtained the opposite results after knockdown of SNHG20 (Fig. 3A-B). Subsequently, the direct interaction between SNHG20 and miR-148a, or between miR-148a and ROCK1 was identified by dual luciferase reporter assay (Fig. 3C-F). We found that the relative luciferase activity in SNHG20-WT or ROCK1-WT group was remarkably suppressed by miR-148a mimics, whereas the relative luciferase activity in SNHG20-MUT or ROCK1-MUT group was not affected (Fig. 3 E&F). Additionally, a significant higher level of ROCK1 was found in OC cells in comparison with normal ovarian epithelial cells (Fig. 3G-H). Therefore, SNHG20 modulated the expression of ROCK1 through targeting miR-148a in OC cells.

### Knockdown of SNHG20 suppressed the proliferation and promoted apoptosis of OC cells through up-regulating miR-148a

**Fig. 4:**
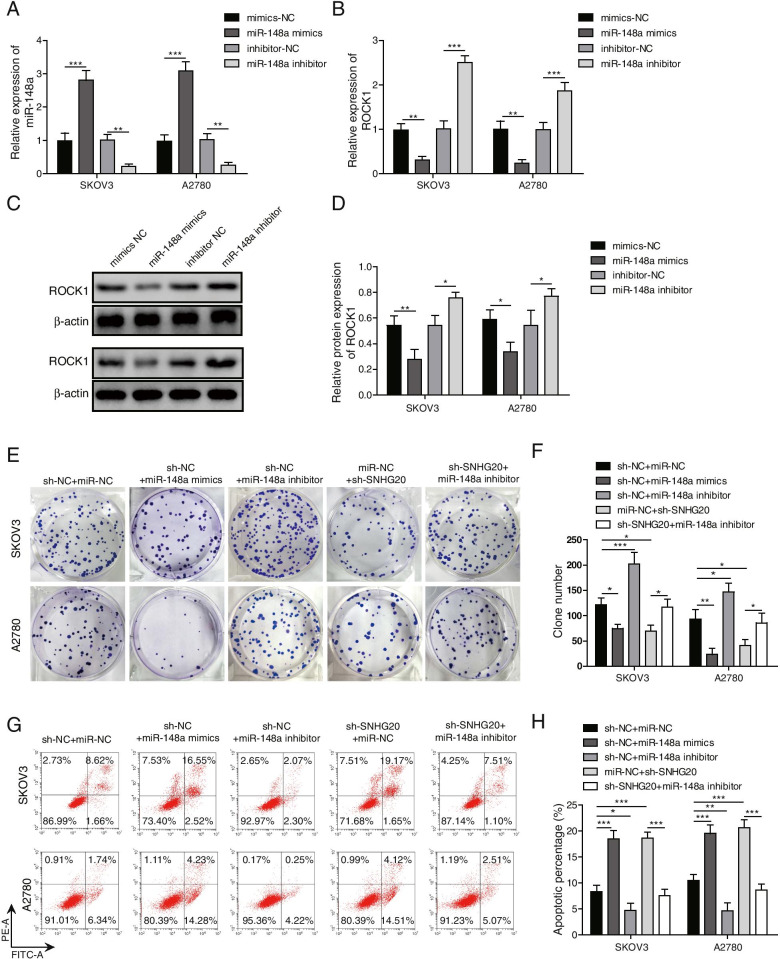
Knockdown of SNHG20 suppressed the proliferation and promoted apoptosis of OC cells via up-regulating miR-148a. (**A**) The expression of miR-148a was measured by qPCR after transfection with miR-148a mimics or miR-148a inhibitor; (**B**) The mRNA expression of ROCK1 was measured by qPCR after transfection with miR-148a mimics or miR-148a inhibitor; (**C**) The protein expression of ROCK1 was detected by western blotting after transfection with miR-148a inhibitor or miR-148a mimics; (**D**) Quantitative analysis of the protein bands; (**E**) Cell proliferation was assessed by colony formation assay; (**F**) Quantification analysis of cell proliferation after transfection with sh-SNHG20 and miR-148a inhibitor; (**G**) Cell apoptosis was detected by flow cytometry; (**H**) Quantification analysis of cell apoptosis after transfection with sh-SNHG20 and miR-148a inhibitor. *P < 0.05, **P < 0.01, ***P < 0.001

To investigate the regulatory function of SNHG20/miR-148a axis in OC, we transfected miR-148a inhibitor or miR-148a mimics into OC cells. The expression of miR-148a was significantly enhanced by miR-148a mimics, while markedly decreased by miR-148a inhibitor (Fig. 4A). In addition, the effect of miR-148a inhibitor or miR-148a mimics on the protein and mRNA expression of ROCK1 was investigated. Knockdown of miR-148a markedly elevated the protein and mRNA levels of ROCK1, whereas overexpression of miR-148a restrained ROCK1 expression (Fig. 4B-D). Functional experiments showed that miR-148a mimics could significantly inhibit OC cell proliferation and induce apoptosis (Fig. 4E-H), while miR-148a inhibitor remarkably promoted cell proliferation and suppressed apoptosis (Fig. 4E-H). Moreover, miR-148a inhibitor could reverse sh-SNHG20-mediated proliferation inhibition and apoptosis promotion in OC cells (Fig. 4E-H). These data suggested that depletion of SNHG20 restrained the proliferation and induced apoptosis of OC cells through targeting miR-148a.

### Down-regulation of SNHG20 suppressed invasion, migration and EMT of OC cells by up-regulating miR-148a

**Fig. 5:**
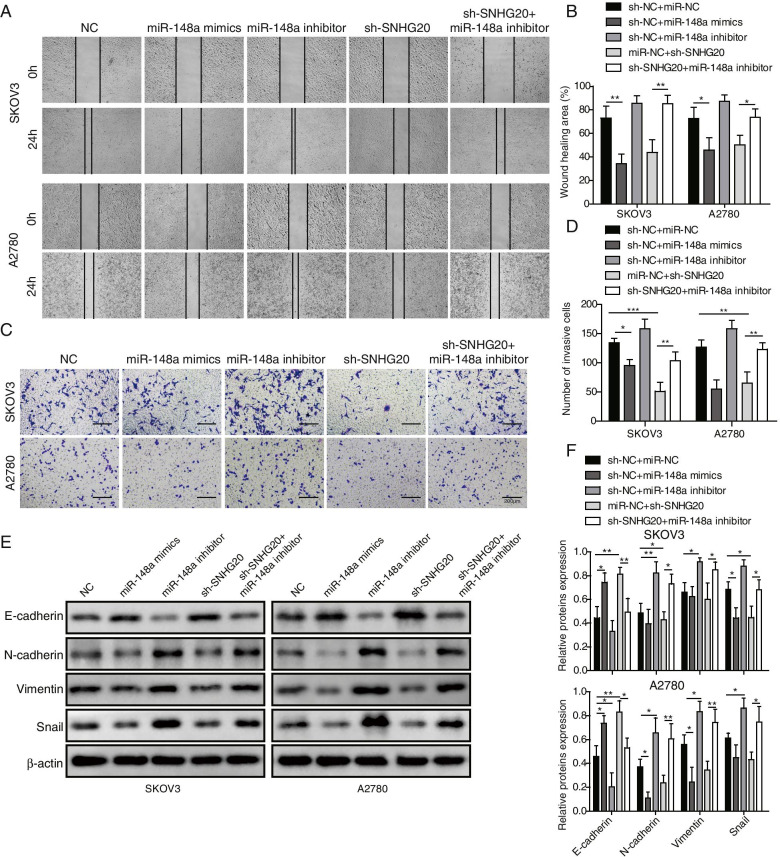
Down-regulation of SNHG20 suppressed invasion and migration of OC cells by up-regulating miR-148a. (**A**) Cell migration was detected by wound healing assay; (**B**) Quantification analysis of cell migration after transfection with sh-SNHG20 and miR-148a inhibitor; (**C**) Cell invasion was measured by transwell assay; (**D**) Quantification analysis of cell invasion after transfection with sh-SNHG20 and miR-148a inhibitor; (**E**) Western blotting was used to determine the expression of EMT-related proteins; (**F**) Quantitative analysis of the protein bands. *P < 0.05, **P < 0.01, ***P < 0.001

Next, the influence of SNHG20/miR-148a axis on OC cell invasion and migration was further determined. miR-148a mimics could significantly inhibit the invasion and migration of OC cells. Conversely, miR-148a inhibitor increased the invasive and migratory abilities of OC cells (Fig. 5A-D). Meanwhile, sh-SNHG20-mediated suppression of invasion and migration of OC cells could be remarkably counteracted by miR-148a inhibitor (Fig. 5A-D). Therefore, SNHG20 might affect OC cell migration and invasion through targeting miR-148a. The invasion and migration of cancer cells have close relationship with EMT process, thus the effect of SNHG20 and miR-148a on the expression of EMT-related proteins was measured. The protein levels of N-cadherin, Vimentin, and Snail were significantly reduced by sh-SNHG20 or miR-148a mimics, but up-regulated by miR-148a inhibitor (Fig. 5E-F). Besides, the regulatory function of sh-SNHG20 on E-cadherin, N-cadherin, Vimentin, and Snail expression could be remarkably reversed by miR-148a inhibitor (Fig. 5E-F). These findings further confirmed that SNHG20 silencing suppressed migration, invasion and EMT of OC cells via regulating miR-148a.

### Down-regulation of SNHG20 inhibited the tumor growth in vivo

**Fig. 6:**
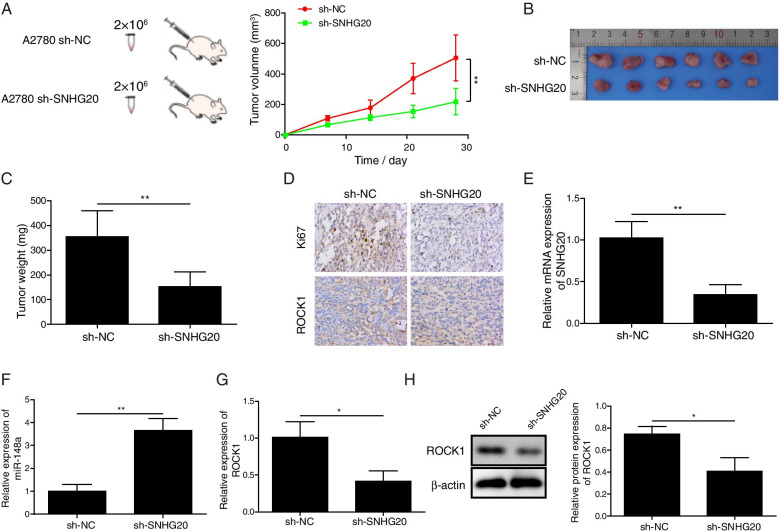
Down-regulation of SNHG20 inhibited the tumor growth in vivo. (**A**) sh-SNHG20 remarkably suppressed the tumor growth; (**B**) Representative pictures of tumors in vivo; (**C**) Measurement of tumor weight; (**D**) Measurement of ki67 and ROCK1 expression in the tumor tissues using immunohistochemical staining; The levels of SNHG20 (**E**), miR-148a (**F**), ROCK1 (**G**) in tumor tissues were assessed by qPCR; (**H**) The protein expression of SNHG20 in tumor tissues was evaluated by western blotting. *P < 0.05, **P < 0.01, ***P < 0.001

**Fig. 7:**
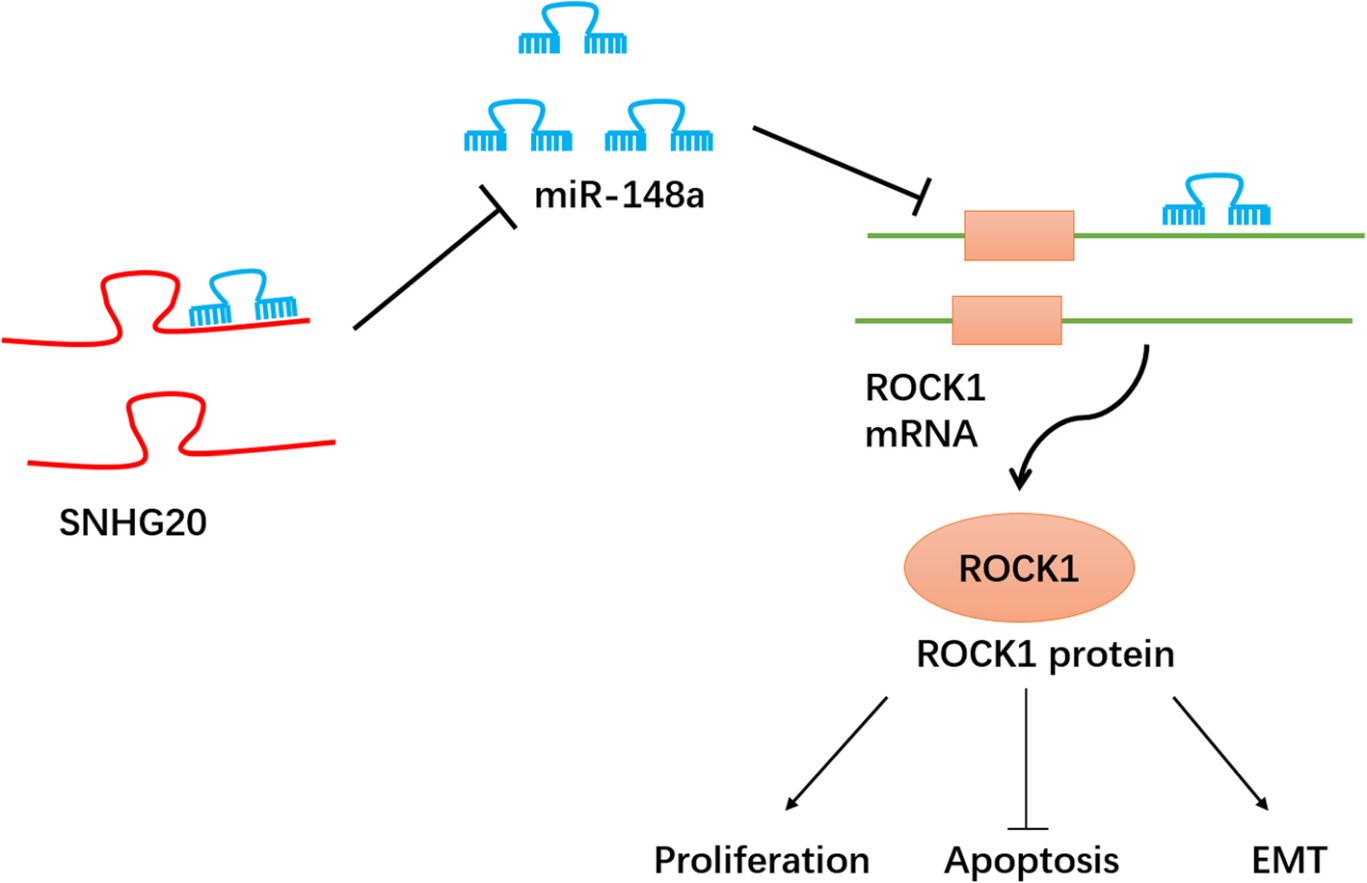
A Schematic Figure of the SNHG20/miR-148a/ROCK1 Axis

In order to further validate the regulatory role of SNHG20 in OC growth in vivo, we conducted the tumor xenograft assay. We found that the tumor growth was markedly inhibited by SNHG20 silencing (Fig. 6A-C). In addition, the expression of ki67 and ROCK1 was measured using immunohistochemical staining (Fig. 6D). The expression of ki67 and ROCK1 was significantly suppressed in the tumor tissues formed by sh-SNHG20-transfected OC cells. Additionally, the expression of SNHG20 and ROCK1 was remarkably suppressed, but the expression of miR-148a was strikingly increased in the tumor tissues from sh-SNHG20 group (Fig. 6E-H). Therefore, SNHG20 depletion could repress the in vivo tumor growth of OC through modulating miR-148a/ROCK1 axis. Therefore, according to the in vivo and in vitro results, a schematic figure (Fig. 7) was established to explain the role of up-regulation of SNHG20 enhanced ROCK1 expression by competitively binding miR-148a, which promoted OC cells EMT and proliferation was established to explain the role of up-regulation of SNHG20 enhanced ROCK1 expression by competitively binding miR-148a, which promoted OC cells EMT and proliferation.

## Discussion

The incidence rate of OC ranks third in the female reproductive system malignant tumors, and the mortality rate ranks first among gynecologic malignancies [22]. The incidence rate of OC in developed countries is about 9.1/10 million, and 10% of OC patients are associated with genetic mutations. The standard treatment for OC is surgery supplemented by postoperative chemotherapy [23]. Although the treatment has been greatly improved, the incidence and mortality rates are still high. Understanding the pathogenesis of OC will contribute to the development of new effective therapies.

Several members of lncRNAs have been confirmed to participate in the progression of cancers through regulating invasion, migration, proliferation, and apoptosis of cancer cells [24, 25]. Meanwhile, a number of lncRNAs have been identified as biomarkers of tumors [26, 27]. Up-regulation of SNHG20 has been validated in several kinds of tumors, including non-small-cell lung cancer, colorectal cancer, gastric cancer, and bladder cancer [3, 15, 28]. In line with these studies, we found a remarkable higher expression of SNHG20 in OC cell lines. Additionally, the invasion, migration, proliferation, and apoptosis inhibition of OC cells were promoted by SNHG20 overexpression in the present study. These data demonstrated that the abnormal high expression of SNHG20 accelerated OC progression.

The involvement of miRNAs in the progression of tumors has been widely investigated. miRNAs might affect the development of various malignancies through regulating the malignant phenotypes of tumor cells [29]. miR-148a has been reported to play a tumor suppressive role in several types of tumors. For example, miR-148a has been documented to function as a tumor suppressor in pancreatic cancer, gastric cancer, and breast cancer through targeting ErbB3 [30], STAT3 [31], and CCK-BR [32], respectively. However, the regulation of miR-148a in OC remains obscure. In the present study, SNHG20 regulated miR-148a expression in OC cells. LncRNAs may regulate the progression of cancer via the competing endogenous RNA (ceRNA) network that lncRNAs and mRNAs reciprocally regulate expression of each other by competitive binding miRNAs [33]. For instance, MCM3AP-AS1 sponged miR-148a, thereby increasing SCLC cell invasion and migration via upregulating ROCK1 expression [34]. In this study, we investigated the relationship between SNHG20 and miR-148a during the development of OC. The direct binding between SNHG20 and miR-148a was confirmed. Additionally, we found that miR-148a inhibitor partly reversed the anti-tumor roles of down-regulation of SNHG20 in OC cells. Therefore, SNHG20 silencing suppressed proliferation, invasion, migration and EMT of OC cells by targeting miR-148a.

It has been documented that ROCK1 favored tumor development via enhancing the invasive and motility abilities and promoting EMT of tumor cells [35, 36]. EMT plays a key role in regulating cancer metastasis, and the altered expression of EMT-related proteins has been considered as a predictor of tumor metastasis [37]. For instance, tumor metastasis is characterized by increased expression of mesenchymal-like phenotype marker vimentin, and lose of epithelial marker such as E-cadherin [38, 39]. As reported, miR-148a acted as a novel suppressor of EMT in NSCLC cells by modulation of ROCK1 expression [35]. Furthermore, miR-148a enhanced the inhibitory effects of sevoflurane on proliferation, invasion and migration and apoptosis inhibition of HCC cells through targeting ROCK1 [34]. Consistently, in this study ROCK1 was identified as a target gene of miR-148a in OC cells. Besides, we found that SNHG20 could influence EMT process of OC cells through targeting miR-148a. Previous studies indicated that ROCK1 could promote the malignant development of lung cancer cells via PTEN/PI3K/FAK pathway [40]. Therefore, ROCK1 may also exaggerate the process of OC through PTEN/PI3K/FAK pathway, which needs to be further investigated. Collectively, SNHG20/miR-148a/ROCK1 axis participated in the pathogenesis of OC cells.

## Conclusions

In summary, the increased expression of SNHG20 facilitated proliferation, migration, invasion, EMT, while induced apoptosis of OC cells. SNHG20 functioned as a ceRNA sponging miR-148a to enhance ROCK1 expression in OC cells. Additionally, we proved that SNHG20 regulated the progression of OC through targeting miR-148a/ROCK1 axis. Therefore, targeting SNHG20/miR-148a/ROCK1 axis might be a promising therapeutic strategy for OC.

## Methods

### Cell culture

Normal human ovarian epithelial cell line and ovarian cancer cell lines (SKOV3, OVCAR-3, CAOV-3, and A2780) were purchased from the American Type Culture Collection (ATCC, Manassas, USA). Cells were cultured in DMEM medium containing 5% FBS with 5% CO_2_ at 37 °C.

### Cell transfection

Short hairpin RNA probed to SNHG20 (sh-SNHG20), pcDNA-SNHG20, miR-148a inhibitor, and miR-148a mimics were obtained from GenePharma (Shanghai, China). Cells were seeded onto 60-mm dishes (Corning, USA) and cultured for 36 h to reach 70% confluence. Lipofectamine 2000 (11,668,500, Invitrogen, CA, USA) was applied for cell transfection. Transfection efficiency was evaluated using qRT-PCR.

### Wound healing test

Cells (4 × 10^5^) were seeded into 6-well dishes. After 70% cell confluence was reached, a pipette tip (200 μL) was used to make a straight scratch with the same width in the middle of the well, followed by washing with phosphate buffer solution (PBS) and replacing with new medium without FBS. After incubation with 5% CO_2_ at 37 °C for 24 h, the width of scratching lines was measured, and cell migration was quantified.

### Transwell assay

Cell invasion was measured using polycarbonate membrane boyden chambers. 1 × 10^4^ cells were seeded onto the top chamber, and the lower chamber was added with 2 mL medium containing 5% FBS. After incubation for 24 h, a cotton swab was used to remove cells on the top surface of membrane. The invasive cells were fixed with 4% paraformaldehyde and stained with 0.3% crystal violet for 20 min. Finally, the invasive cells were captured from at least 3 random fields.

### Dual luciferase reporter assay

Starbase database (http://starbase.sysu.edu.cn/) was applied for predicting the binding sites between miR-148a and SNHG20. TargetScan database (http://www.targetscan.org/vert_72/) showed that miR-148a possessed binding sites in ROCK1 3 ‘UTR. Then luciferase reporter assay was conducted to confirm the relationship between SNHG20/ROCK1 and miR-148a. The wild type (WT) and mutant type (MUT) of ROCK1 3’ UTR or SNHG20 containing miR-148a binding sites were amplified by PCR and cloned into pMIR-REPORT (AM5795, Thermo, USA). Cells were transfected with WT-ROCK1–3′ UTR, MUT-ROCK1–3′ UTR, WT-SNHG20, MUT-SNHG20 reporter plasmid together with miR-148a mimics or miR-NC using lipofectamine 2000. Dual-luciferase reporter gene assay kit (E1910, Promega, USA) was used to detect renilla luciferase and firefly luciferase activity. The firefly luciferase activity was normalized to renilla luciferase activity.

### Apoptosis assay

Annexin V apoptosis measurement Kit (559,763, BD, NJ, USA) was applied to measure apoptosis as previously described [41]. Briefly, 5 × 10^5^ cells were seeded into 6-well plates, and cultured in the incubator. After different treatments, the cells were washed three times using cold PBS, and resuspended in 200 μL of buffer containing 10 μL of Annexin V-FITC. Cells were incubated at 4 °C in the dark for 5 min and apoptosis was analyzed by flow cytometry.

### RNA isolation and real-time PCR

Total RNA was extracted with the mirVana™ PARIS™ Kit (AM1556, Ambion, TX, USA) as described [42]. The purity of RNA was determined by micro ultraviolet spectrophotometer (Shimadzu international Co. Ltd. Shanghai, China). The RNA was reversely transcribed into cDNA with SuperScript TM II Reverse Transcriptase (18080–200, Invitrogen, USA). RT-qPCR was carried out using SYBR@ Premix Ex TaqTM (DR100AM, TaKaRa, Beijing, China). All samples were detected in triplicate for each specific gene. The cycle threshold values for each gene were normalized to control gene. The primers used were presented as follows. SNHG20: forward: 5′-CAGGGCATCCAGGTCAGTTT-3′ and reverse: 5′-AGTATGTGGTCACCTTGGCG-3′; ROCK1: forward: 5′-TGCAACTGGAACTCAACCAA-3′ and reverse: 5′-GTTTAGCACGCAATTGCTCA-3′; miR-148a: forward: 5′- CGGCTCAGTGCACTACAGAA-3′ and reverse: 5′- GTCGTATCCAGTGCAGGGTCCGAGGTATTCGCACTGGATACGAC ACAAAG-3′; GAPDH: forward: 5′-CCAGGTGGTCTCCTCTGA-3′ and reverse: 5′-GCTGTAGCCAAATCGTTGT-3′; U6: forword: 5′-CTCGCTTCGGCAGCACA-3′ and reverse: 5′-AACGCTTCACGAATTTGCGT-3′.

### Western blotting

Western blotting was performed as described previously [43]. All primary antibodies were purchased from Abcam (Cambridge, UK). Total protein lysates were prepared using lysis buffer containing 1% PMSF. Concentration of protein was measured with a BCA protein assay kit (CW0014, CWBIO, China). Same amount of protein was loaded onto SDS-PAGE and then transferred to PVDF membranes (IPFL00010, Sigma-Aldrich, USA). The membranes were blocked in 5% non-fat milk and incubated with primary antibody at 4 °C overnight. After washing for three times, the membranes were incubated with secondary antibody in TBST for 2 h at room temperature. Proteins were measured with an enhanced chemiluminescence detection kit (Thermo Fisher Scientific Inc.). Image J software was used for quantitative analysis of protein band density. The primary antibodies used in this study are as follow: anti-ROCK1 (1:1000, #4035, Cell Signaling Technology, USA), anti-E-cadherin (1:1000; #14472, Cell Signaling Technology), anti-Vimentin (1:1000; #3932, Cell Signaling Technology), anti-N-cadherin (1:1000; #4061, Cell Signaling Technology) anti-Snail (1:1000; #3879, Cell Signaling Technology) and anti-β-actin (1:1000, #4970, Cell Signaling Technology).

### Colony formation test

Cells (1 × 10^3^ per well) were plated into 6-well dishes and cultured on the condition of 5% CO_2_ at 37 °C for 14 d. After the removal of media, cells were washed three times with PBS and stained with crystal violet for 1 h. Quantity One software (Bio-Rad, Richmond, USA) was used to quantify colonies (> 40 μm). The results were presented as an average of three independent measurements.

### Tumor xenograft assay

The in vivo tumor growth was detected according to a previous study [18]. BALB/C-nu/nu nude mice (male, 8 weeks, 18–20 g) were purchased from Hunan SJA Laboratory Animal Co., Ltd. (Changsha, China) and kept in specific pathogen free (SPF) environment of China-Japan Union Hospital of Jilin University. All the animal experiments were approved by the ethical committee of China-Japan Union Hospital of Jilin University. A total of 12 mice were randomly divided into two groups (*n* = 6 per group). The A2780 cells were stably transfected with pLP/VSVG-sh-SNHG20 plasmid or blank pLP/VSVG vector (sh-NC) and then subcutaneously injected into the dorsal flank of mice. After tumor implantation for 8 weeks, the mice were euthanized by cervical dislocation, and tumor tissues were photographed and weighed. The tumor volume was quantified with the formula V (mm^3^) = 0.5 × a × b^2^ (a is the maximum length to diameter, and b is the maximum transverse diameter).

### Immunohistochemistry staining

Immunohistochemistry staining was conducted as previously reported [44]. The collected tumor tissues were fixed in 5% formalin for 24 h, and embedded in OCT compound. Then, tissues were sectioned into 10-μm thickness. After antigen retrieval using microwave oven, the sections were washed twice (5 min/time) incubated in 10% H_2_O_2_ for 5 min. After washing twice (5 min/time), the sections were blocked in 5% goat serum. Primary antibodies anti-ROCK1 (1:100, ab45171, Abcam, UK) and anti-ki67 (1:500, ab15580, Abcam) were applied at 4 °C overnight. Then, the sections were incubated with secondary antibody for 2 h. DAB reagent was added to the sections for developing, and the images were captured under an inverted microscope (Olympus BX41, Tokyo, Japan).

### Fluorescence in situ hybridization (FISH)

The FISH probes and FISH assay were performed according to the protocol of Stellaris RNA FISH (Bioscience Technologies). Briefly, cells were fixed in 5% formaldehyde for 5 min and washed three times using PBS, followed by incubation in 70% ethanol at 37 °C for 2 h. Then cells were incubated in 10% deionized formamide for 3 min. Subsequently, probes were prepared using hybridization buffer (0.2 g/mL dextran sulfate, 15% deionized formamide), and cells were incubated with the probes in 2× saline-sodium citrate for 20 h at 37 °C in the dark. Then, cells were maintained in 10% deionized formamide for 1 h at room temperature. After washing three times with 2× saline-sodium citrate, images were captured using a confocal microscopy (LSM510, Zeiss).

### Statistical analysis

All experiments were conducted at least for 3 times. Results were shown as mean ± SD. Student’s t test was applied between two groups. Multiple comparisons were conducted using one-way analysis of variance (ANOVA) followed by Tukey post hoc test. A *P* value < 0.05 was considered as statistically significant.

## Data Availability

The datasets used or analyzed during the current study are available from the corresponding author on reasonable request.

## Abbreviations

OC: Ovarian cancer
lncRNAs: Long non coding RNAs
miRNAs: microRNAs
SNHG20: small nucleolar RNA host gene 20
miR-148a: microRNA-148a
ROCK1: Rho-kinase1
FISH: Fluorescence in situ hybridization
ANOVA: analysis of variance
